# Editorial: thematic series “Integrating movement ecology with biodiversity research”

**DOI:** 10.1186/s40462-020-00210-0

**Published:** 2020-05-25

**Authors:** Florian Jeltsch, Volker Grimm

**Affiliations:** 1 Plant Ecology and Nature Conservation, University of Potsdam, Am Mühlenberg 3, 14476 Potsdam, Germany; 2 Department of Ecological Modelling, Helmholtz Centre for Environmental Research-UFZ, Permoserstr 15, 04318 Leipzig, Germany

Movement ecology and biodiversity research are distinct subdisciplines of ecology. To make progress in both of them, they need to be better integrated. Movement ecology provides a unifying framework, based on first principles, for studying the movement of organisms. Being launched as a declared discipline only about 10 years ago [17], movement ecology has developed much technology and analytical tools to decipher how animals integrate information about their environment, experience, and innate states to make movement decisions [5, 9]. Still, the focus on individual organisms makes it difficult to address the ecological consequences of movement for populations, communities, and ecosystems. Moreover, putting movement into its broader ecological context could have important repercussions on the framework of movement ecology itself. Similar feedbacks occurred in other fields focusing on individual organisms, where putting individual-level theories into ecological context helped identifying their limitations and hence led to theory refinement. Good examples for such positive feedbacks are provided by ‘energy budget theory’ (e.g. [15]) and ‘optimal foraging theory’ (e.g. [20, 21]).

Biodiversity research has a longer history, with its roots going back to community ecology and biogeography. It explores the emergence, maintenance, and function of diversity at all levels of biological organization. Because of its strong focus on the dynamics and coexistence of species, individuals and their behavior are usually not addressed explicitly. Movement, however, is particularly important to consider for the majority of species, which have low abundances and are thus strongly affected by temporal and spatial heterogeneity and individual interactions. The recently proposed conceptual framework of “coviability” [12] therefore suggests a better integration of individual organism and their behavior into community theory and, hence, biodiversity research. Moreover, also for another key question of biodiversity research, how species composition will change due to range shifts and invasive species, a mechanistic understanding of movement, in particular dispersal, is critical [22]. Hence, correlative species distribution modelling from macroecology needs to be complemented by mechanistic modelling of population dynamics and dispersal (e.g., [19, 30]).

In fact, evidence is accumulating that many of the mechanisms that shape biodiversity are mediated by organismal movement. Movement promotes diversity both directly through species’ own mobility patterns and indirectly through mobile-link functions of moving animals [13]. This includes the important role of animal vectors that transport seeds, pollen, larvae, fungi, bacteria, and even adult organisms. Widely discussed dispersal-related mechanisms affecting biodiversity are mass effects, colonization-competition trade-offs and dispersal limitation [11]. Moreover, movement patterns of organisms can critically influence community assembly and species coexistence in less obvious ways by, for example, reducing exploitation competition in spatiotemporally heterogeneous environments [14], strengthening predator effects on prey [1], or modifying abiotic conditions in critical ways [25]. Despite of this obvious relevance of movement, highly aggregated representations of movement still prevail in biodiversity research, such as dispersal kernels or space-use patterns, which ignore how moving organism actually interact and navigate through heterogeneous habitat.

Biodiversity research has a species, or population perspective, while movement ecology has an individual organism perspective [11, 22]. This gap needs to be filled: Ignoring individuals and their behavior limits progress in our understanding of biodiversity. Likewise, with a sole focus on the movement process itself, movement ecology might contribute less to unifying ecology theory than has been expected from individual-based approaches in general [10].

Bridging the gap between biodiversity research and movement ecology is possible. First integrations demonstrated that individual movement capacities and strategies are critical in determining the persistence of species and communities in fragmented landscapes [3, 7], with changing climatic conditions [27], or in the presence of invasive species [4]. At the same time, the ever-increasing human impact on nature puts long-established movement patterns in jeopardy, and organismal movement is changing perceivably across scales [6, 8, 26, 29]. Yet, a full-fledged integration of movement ecology and biodiversity research is still in its infancy [11]. Empirically, we need more studies that not only focus on the movement of individuals, but also how they interact, while moving, with their environment and with other individuals, including their own and other species. From a theoretical viewpoint, there is a lack of modelling approaches that integrate individual movement and its consequences with population and community dynamics [12].

This thematic series aims to bring together studies that make a step towards the urgently needed integration of movement ecology and biodiversity research. It goes back to an international symposium held under this title in Potsdam, Germany, in September 2018. Organized by the project ‘BioMove’ (‘Integrating Biodiversity Research with Movement Ecology in dynamic agricultural landscapes’, www.biomove.org) presentations and lively discussions of more than 120 participants created a momentum and spirit that, inter alia, led to the initiative for this thematic series.

Three of the six contributions to this Thematic Series directly address multiple species and hence biodiversity. Meyer et al. [16] determine movement corridors for nine large forest-dwelling species of mammals, including tapir, two species of both deer and peccary, jaguar, puma, ocelot and the giant ant-eater. The latter as well as the white-lipped peccary and the tapir were treated as highly sensitive species, while the others were labelled “tolerant”. Two methods were compared: occupancy models based on camera trap data and step-selection functions based on GPS telemetry data. Both methods gave similar results for the tolerant species but not for the sensitive ones. This is one of the first movement ecology studies delineating corridors which addresses a whole suite of species. Recommendations for conservation are thus more comprehensive and are addressing biodiversity issues more directly. The increasing availability of geo-referenced presence and movement data will enable similar approaches of other species and communities in the future.

Bielčik et al. [2] review an important type of movement that so far has largely been ignored in movement ecology: hyphae-mediated movement in filamentous fungi. Recent advances in fungal ecology on topics like informed growth, mycelial translocations, or fungal highways have not yet been linked to theoretical developments within movement ecology. To better integrate mycology and movement ecology, the authors introduce the concept of “active movement in filamentous fungi”, defined as “the translocation of biomass within the environment brought about by the organism’s own energy resources.”

Schuppenhauer et al. [23] explore passive movement of soil-dwelling arthropods, springtails (Collembola) and moss mites (Oribatida), which play a key role for the ecosystem functions and services provided by soils. Their active movement is too limited to contribute to colonizing new soils. Passive movement via wind, other animals or sea currents has been studied before, but not yet for running waters. Using field and lab experiments the authors found that dispersal abilities of moss mites in terms of submersion survival and floating ability are high but also species specific. They conclude that running waters provide important and effective dispersal highways for many of these soil-living species.

The three other contributions to the Thematic Series focus on the movement of single species but address question that are highly relevant to biodiversity research. Seidel et al. [24] link high-resolution movement data of the Namibian black rhino to satellite data about habitat productivity. They estimated the recursion movement of 59 individuals by investigating patterns in 24-h displacement at different times of the day for intermediate-scale distances and daily for larger distances. Short-term recursion was highest for areas of median, not highest productivity. Rhinos stayed within the same area within their home ranges for several days, but recursion along larger time scales was observed as well and is likely to contribute to maintaining open landscapes and savannas.

The two remaining contributions address evolutionary aspects. Wolz et al. [28] compare traits characterizing dispersal and reproduction of a predatory wasp spider in its core populations (Southern France) and those in Baltic States to where the species‘range expanded over the recent decades. The question was whether this range expansion was related to evolutionary changes in dispersal. This was not the case, but differences were observed in the response of dispersal to winter conditions, i.e. increased ballooning for long-distance movement after winter conditions matching those in native habitats and decreased ballooning under mis-matching conditions. The authors interpret these differences in terms of intergenerational plasticity rather than as an evolutionary response.

Premier et al. [18] explore how, in highly fragmented landscapes, landscape structure and movement syndromes (“shy” vs. “bold”) interact in determining local and regional genetic diversity. They used the European lynx as an example and added neutral genetic markers to an existing individual-based model. “Bold” dispersers, who spend more time in matrix habitat, can “save” genetic diversity because they are more likely to reach other habitats, but also decrease genetic diversity because as founders in previously unoccupied habitats they may prevent establishing other genotypes. “Shy” dispersers, on the other hand, maintain a more gradual genetic drift. These findings have implications for reintroduction and reinforcement projects, which should take diversity in movement behaviour, expressed by different animal personalities, into account. In this context, well-tested individual-based population models, augmented by genetic aspects, are suitable tools.

As these different contributions show, the claimed integration can be approached from different angles, ranging from a more applied conservation focus to more basic research on species interactions and community dynamics. We welcomed both bottom-up and top-down approaches, i.e. movement studies relating their design and/or findings to biodiversity, and biodiversity studies, which related their design and/or findings to the movement of organisms. In either case, the common thread is provided by bringing movement and biodiversity dynamics in a common context. As discussed and exemplified by Jeltsch et al. [11] and Schlägel et al. [22], the integration of movement ecology and biodiversity research is challenging but also promising, leading to insights than can help us to better understand how biodiversity emerges, is maintained, and can be protected and restored.
